# Spontaneous breathing with airway pressure release ventilation favors ventilation in dependent lung regions and counters cyclic alveolar collapse in oleic-acid-induced lung injury: a randomized controlled computed tomography trial

**DOI:** 10.1186/cc3908

**Published:** 2005-11-16

**Authors:** Hermann Wrigge, Jörg Zinserling, Peter Neumann, Thomas Muders, Anders Magnusson, Christian Putensen, Göran Hedenstierna

**Affiliations:** 1 Assistant Professor, Department of Anaesthesiology and Intensive Care Medicine, University of Bonn, Sigmund-Freud-Strasse 25, D-53105 Bonn, Germany and Research Fellow, Department of Clinical Physiology, University of Uppsala, University Hospital, SE-751 85 Uppsala, Sweden; 2 Physicist and Research Associate, Department of Anaesthesiology and Intensive Care Medicine, University of Bonn, Sigmund-Freud-Strasse 25, D-53105 Bonn, Germany; 3 Assistant Professor, Department of Anaesthesiology and Intensive Care Medicine, University of Göttingen, Robert Koch Strasse 40, D-37075 Göttingen, Germany; 4 Resident, Department of Anaesthesiology and Intensive Care Medicine, University of Bonn, Sigmund-Freud-Strasse 25, D-53105 Bonn, Germany; 5 Professor of Radiology, Department of Radiology, University of Uppsala, University Hospital, SE-751 85 Uppsala, Sweden; 6 Professor of Anaesthesiology and Intensive Care Medicine, Department of Anaesthesiology and Intensive Care Medicine, University of Bonn, Sigmund-Freud-Strasse 25, D-53105 Bonn, Germany; 7 Professor of Clinical Physiology, Department of Clinical Physiology, University of Uppsala, University Hospital, SE-751 85 Uppsala, Sweden

## Abstract

**Introduction:**

Experimental and clinical studies have shown a reduction in intrapulmonary shunt with spontaneous breathing during airway pressure release ventilation (APRV) in acute lung injury. This reduction was related to reduced atelectasis and increased aeration. We hypothesized that spontaneous breathing will result in better ventilation and aeration of dependent lung areas and in less cyclic collapse during the tidal breath.

**Methods:**

In this randomized controlled experimental trial, 22 pigs with oleic-acid-induced lung injury were randomly assigned to receive APRV with or without spontaneous breathing at comparable airway pressures. Four hours after randomization, dynamic computed tomography scans of the lung were obtained in an apical slice and in a juxtadiaphragmatic transverse slice. Analyses of regional attenuation were performed separately in nondependent and dependent halves of the lungs on end-expiratory scans and end-inspiratory scans. Tidal changes were assessed as differences between inspiration and expiration of the mechanical breaths.

**Results:**

Whereas no differences were observed in the apical slices, spontaneous breathing resulted in improved tidal ventilation of dependent lung regions (*P *< 0.05) and less cyclic collapse (*P *< 0.05) in the juxtadiaphragmatic slices. In addition, with spontaneous breathing, the end-expiratory aeration increased and nonaerated tissue decreased in dependent lung regions close to the diaphragm (*P *< 0.05 for the interaction ventilator mode and lung region).

**Conclusion:**

Spontaneous breathing during APRV redistributes ventilation and aeration to dependent, usually well-perfused, lung regions close to the diaphragm, and may thereby contribute to improved arterial oxygenation. Spontaneous breathing also counters cyclic collapse, which is a risk factor for ventilation-associated lung injury.

## Introduction

Spontaneous breathing in any phase of the mechanical ventilator cycle is possible during airway pressure release ventilation (APRV), a technique that provides ventilatory support by time-cycled switching between two continuous positive airway pressure levels [1-3]. Studies in patients with acute lung injury (ALI) suggest that spontaneous breathing with APRV not only reduces the need for sedation to adapt the patient to the ventilator [4], but also improves both systemic blood flow and arterial blood oxygenation when compared with controlled mechanical ventilation [4-6]. Mechanisms for improved oxygenation with spontaneous breathing during APRV include a reduction in intrapulmonary shunting and improved ventilation-perfusion matching, as demonstrated in experimental and clinical studies [6-9]. In a recent computed tomography (CT) study in pigs with oleic-acid-induced lung injury, we observed recruitment of the atelectatic lung and an increased end-expiratory lung volume (EELV) by spontaneous breathing [10]. This should reasonably contribute to reduce shunting.

Ventilation at lower EELV and/or low levels of positive end-expiratory pressure (PEEP) in the absence of spontaneous breathing may also cause cyclic opening of lung units during inspiration (tidal recruitment) and closing of lung units during expiration. Cyclic opening and closing of lung regions causes shear stress that may contribute to ventilator-induced lung injury [11].

Controlled mechanical ventilation in the supine position causes larger tidal excursions of the diaphragm in its upper, anterior part than in its dorsal segment [12]. This is due to higher abdominal pressure in the dependent regions that limits diaphragmatic movement more there than in nondependent regions [12]. On the other hand, posterior muscular sections of the diaphragm move more than the anterior tendon plate during spontaneous breathing [13]. Mechanical ventilation is consequently distributed preferentially to the anterior regions while dependent regions are favored during spontaneous breathing [12,14]. Based on these studies [13,15], we hypothesized that spontaneous breathing with APRV favors the distribution of intrapulmonary gas to dependent, usually well-perfused, lung regions, making them better aerated than in subjects with a passively moving diaphragm. Moreover, we assumed that with better aeration of dependent lung regions there may be less cyclic collapse (or tidal recruitment) of lung tissue. This should reduce the shear stress caused by the opening and closing of air spaces. These hypotheses were tested using dynamic (high time resolution) CT scanning of single apical and juxtadiaphragmatic lung regions during tidal ventilation in porcine oleic-acid-induced ALI.

## Materials and methods

### Experimental setting and protocol

The study was performed in the research laboratory of the Department of Clinical Physiology at the University Hospital of Uppsala, Sweden and was approved by the local institutional review board for animal studies. Dynamic CT scans were performed in 22 pigs with oleic-acid-induced lung injury. Pigs have also been included in a different CT study addressing effects of spontaneous breathing on end-expiratory atelectasis and lung volume using spiral CT scans of the total lung during end-expiratory clamping [10]. The present study shows regional analyses of dynamic CT scans obtained during sustained tidal ventilation as described in the following. The animal preparation has been described in detail previously [10]. Briefly, anesthesia was induced intramuscularly and maintained by infusions of 30 mg/kg/hour ketamine, 0.1 mg/kg/hour midazolam, and 1–2 μg/kg/minute remifentanil. The pigs were mechanically ventilated via a tracheostomy in the supine position and lung injury was induced with repeated central venous oleic acid injections (0.1 ml/kg) as described earlier [10]. Two hours after the induction of ALI, animals were randomized using sealed envelopes to receive either APRV with or without spontaneous breathing. Six hours after randomization, the pigs were transferred to the radiology department without interrupting ventilation or changing the ventilatory mode, and transverse dynamic chest CT scans were then performed.

### Ventilatory setting

#### APRV without spontaneous breathing

Time-cycled pressure-controlled ventilation (Evita 4; Dräger, Lübeck, Germany) was applied with a respiratory rate of 20 breaths/minute, an inspiratory:expiratory time ratio of 1:1, FiO_2 _of 0.5, PEEP of 5 cmH_2_O, and an inspiratory pressure that resulted in a tidal volume (V_T_) of approximately 10 ml/kg. Adjustments of the respiratory rate up to 30 breaths/minute and increments of inspiratory pressure were allowed in order to avoid hypercapnia above a PaCO_2 _of 60 mmHg and to compensate for decreased compliance. To suppress spontaneous breathing during hypercapnia, the remifentanil infusion was increased to 2 μg/kg/minute and, if spontaneous breathing efforts were detected in esophageal pressure and/or gas flow tracings, 2.5 mg/hour pancuronium bromide was infused for muscle relaxation (this was necessary in four animals). The PEEP, the inspiratory:expiratory ratio, and FiO_2 _were kept constant during the entire study.

#### APRV with spontaneous breathing

The ventilator settings were guided by the principles already described. To allow reinstitution of spontaneous breathing the respiratory rate of the ventilator was decreased to 15 breaths/minute, resulting in (preset) inspiratory and expiratory times of 2 s, and the remifentanil infusion was lowered.

### Gas analysis, ventilatory, and lung mechanics measurements

Methods and techniques for gas analysis, ventilatory, and lung mechanics measurements have been described elsewhere [10].

### CT scanning and analysis

During ventilation a frontal topogram of the chest was obtained with a Somatom Plus 4 (Siemens, Erlangen, Germany) and two transverse slices with a thickness of 8 mm were defined: one apical slice located at the midpoint of the intrathoracic trachea, and another slice located 1–2 cm above the diaphragm.

In each of the two defined transverse slices, dynamic CT scans (140 kV, 111 mA, 0.75 s for one spin) over an acquisition time of 4.5 s were performed without interrupting ventilation in random order, ensuring inclusion of at least one ventilatory cycle. The CT scanner provided image reconstructions with effective time resolution of 0.1 s, resulting in 45 single scans for each slice. Depending on the transverse image size, the pixel size was 0.25 ± 0.05 mm^2^, resulting in a voxel size of 1.96 ± 0.39 mm^3^.

The CT images were transferred to a personal computer and analyzed with a computer program (Osiris; University of Geneva, Switzerland). The investigator was blinded to the ventilatory mode. In all slices of the dynamic scans of the two defined lung regions, the entire lung was chosen as the region of interest (ROI) by drawing the external boundaries of the lungs inside the ribs and the internal boundaries along the mediastinal organs. The images with the highest mean Hounsfield units (HU) and with the lowest mean HU in the following sequence of images with decreasing HU numbers, representing the end-expiratory and end-inspiratory images (during APRV with spontaneous breathing, breaths with combined ventilator and spontaneous breathing activity), were selected by another researcher (JZ) in order not to unblind the ventilatory mode and passed to the investigator (TM) for further analysis.

**Figure 1:**
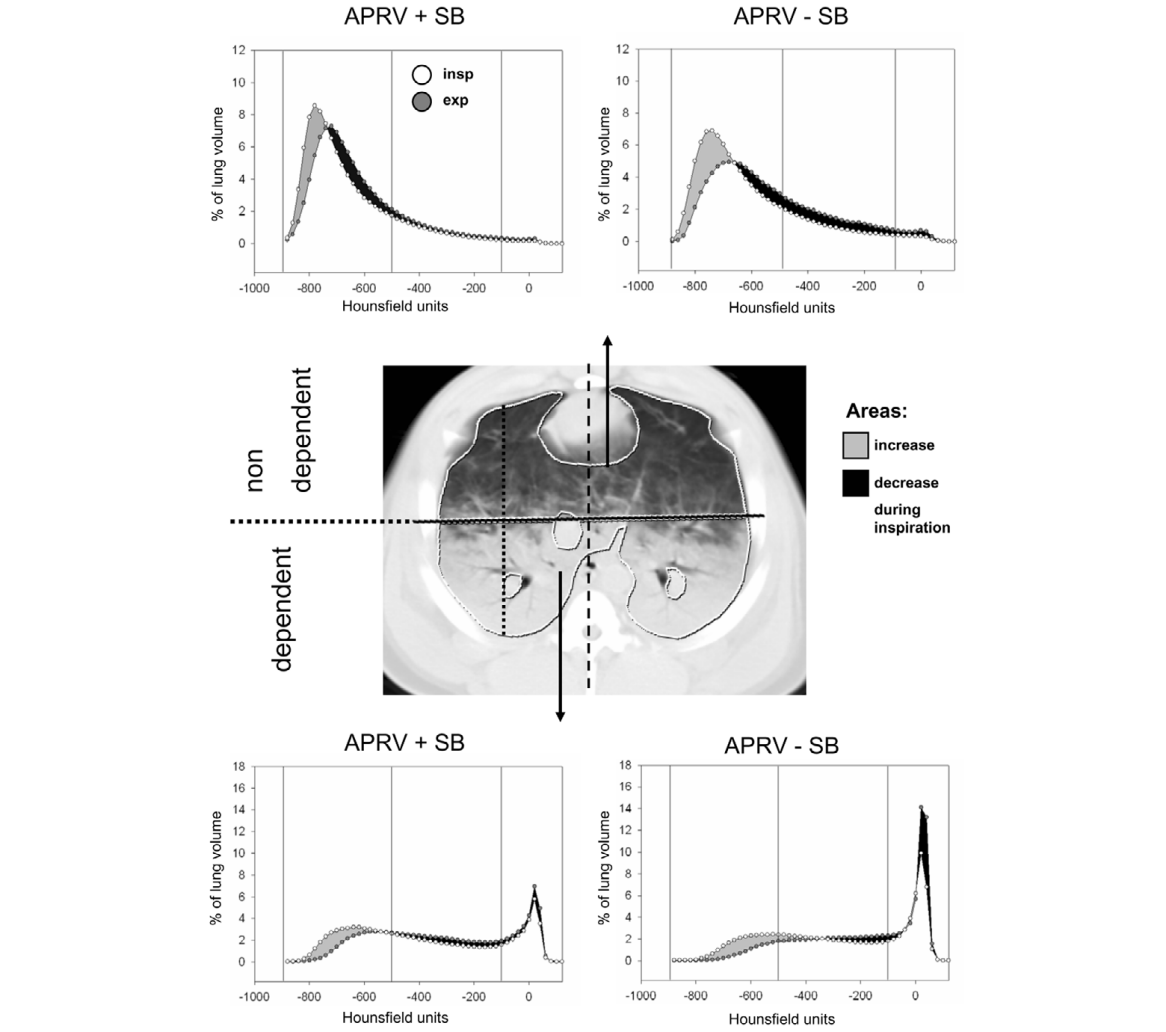
Densitometries of dynamic lung computed tomography (CT). Center, example of an end-expiratory transverse CT scan of a slice close to the diaphragm. External boundaries of the lungs inside the ribs and the internal boundaries along the mediastinal organs were marked. The marked lung tissue was divided into a nondependent and a dependent region of interest bisecting a ventral to dorsal axis that represents the highest chest diameter in parallel to a reference line from sternum to spine. Upper and lower parts, densitometric analyses of all dependent and nondependent lung regions close to the diaphragm computed from dynamic CT at end-expiration and end-inspiration. Histograms show the normalized lung volume for Hounsfield units ranging from -1,000 to 100, plotted as means of all animals. Aeration categories (hyperinflated, normally aerated, poorly aerated, nonaerated; see text for details) are marked. Gray areas indicate increase during inspiration, black areas show decrease during inspiration.

The marked lung was divided into a ventral, nondependent half and a dorsal, dependent half (Figure 1, center) bisecting a ventral to dorsal axis that represents the highest chest diameter in parallel to a reference line from the sternum to the spine. Larger blood vessels were marked separately and data were subtracted. The number of voxels corresponding to each attenuation value in ventral and dorsal ROIs of each slice were counted and stored by the computer program. Attenuation values outside the range of -1,000 to +100 HU, which constituted less than 1% of all counts, were excluded. For repeated marking of the external boundaries of the lungs using similar technology, the intraobserver variability for the determination of ROIs was 1.7% of the mean ROI area [16].

Continuous attenuation distributions of each ROI were analyzed using four groups of attenuation ranges with decreasing air content [17-19]: range I included attenuation values between -1,000 and -900 HU previously defined as hyperinflation, range II included values between -900 and -500 HU defined as normal aeration, range III included values between -500 and -100 HU previously defined as poor aeration, and range IV included values between -100 and +100 HU representing atelectasis or lung parenchyma with a gas content of 10% or less.

The gas content of each ROI was calculated as follows: (mean HU / -1,000) × voxel number × voxel volume. For the calculation of the gas content, all voxels ranging from -1,000 to 0 HU were used.

These calculations were performed to obtain the regional distribution of aeration and the amount of nonaerated lung tissue in end-expiratory scans as well as end-inspiratory scans. To assess the distribution of regional ventilation, the end-expiratory gas volume was subtracted from the gas volume calculated from the end-inspiratory scans. Tidal recruitment (cyclic collapse) was estimated by subtracting the calculated amount of nonaerated tissue in inspiratory scans from the corresponding values in expiratory scans.

### Statistics

Primary outcome measures were the tidal changes in regional gas volumes and the amount of nonaerated lung. Sample size estimation was based on findings from previous studies. To detect differences in these parameters between ventilatory settings with the given two-sided parallel design at a significance level of 5% (α = 0.05) with a probability of 80% (β = 0.20), based on an estimated difference of 1.25 of the parameter's mean standard deviation, at least 20 animals had to be studied.

Results are expressed as the mean ± standard deviation. All statistical analyses were performed using a statistical software package (STATISTICA for Windows 6.0; StatSoft, Inc., Tulsa, OK, USA). A normal distribution of data was confirmed by the Shapiro–Wilks *W *test. Two-way analysis of variance with the factors lung region and ventilator mode was used to detect regional aeration differences. Continuous attenuation distributions were tested using analysis of variance with the factors HU range and ventilatory mode in each ROI. Only when a significant *F *ratio was obtained for the two factors or its interaction were differences separated using Student's *t *test with Bonferoni correction for multiple tests. Differences were considered to be statistically significant if *P *< 0.05.

## Results

### Cardiorespiratory effects

Cardiorespiratory effects have been reported earlier [10] and are provided in condensed tables I-III in an online supplement (see Additional File 1, data kindly reproduced with permission of Lippincott, Williams and Wilkins, Baltimore, MD, USA). No between-groups differences in any measured cardiorespiratory variable have been observed before and 2 hours after the induction of lung injury when pigs were randomized, also indicating that the degree of lung injury was comparable between the groups [10]. During specific ventilatory treatment, apart from a significantly higher effective respiratory rate (including spontaneous breathing) and a trend towards lower tidal volumes with spontaneous breathing, ventilatory variables such as the minute ventilation, alveolar ventilation and airway pressures were comparable between APRV with and without spontaneous breathing (see Additional File 1, tables I-III, data kindly reproduced with permission of Lippincott, Williams and Wilkins, Baltimore, MD, USA) [10]. The proportion of minute ventilation due to spontaneous breathing during APRV with spontaneous breathing could not be measured directly, because the spontaneous breathing activity partially coincides with mechanical breaths. However, the mechanical ventilation should have been halved by the 50% reduction of the respiratory rate of the ventilator down to 15 breaths/minute during APRV with spontaneous breathing. Minute ventilation was comparable between both settings, however, suggesting a 50% contribution of spontaneous breathing to the total ventilation.

### Regional distribution of tidal ventilation and recruitment

Analyses of continuous attenuation distributions of apical and diaphragmatic ROIs during both inspiration and expiration are plotted in Figure 1. The differences between inspiration and expiration are marked.

**Figure 2:**
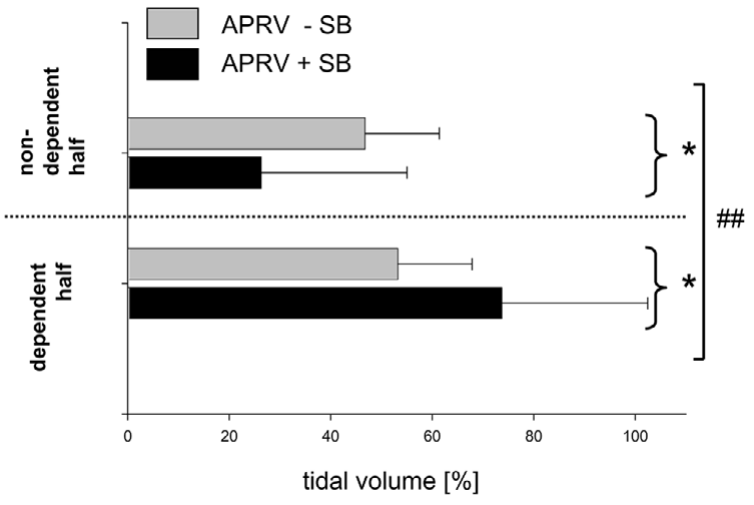
Regional distribution of tidal gas volume. Tidal changes in gas distribution of diaphragmatic slices between a nondependent region of interest (ROI) and a dependent ROI during airway pressure release ventilation (APRV) with or without spontaneous breathing (+SB/-SB). Data presented as the percentage (mean ± standard error of the mean) of the total gas volume of voxels for each ROI. ^##^*P *< 0.01, interaction of factor ROI with factor ventilatory mode, suggesting that the ventilatory mode has a significant influence on the regional distribution of tidal volume. *P < 0.05 between ventilatory groups (*post hoc *results given only if significant).

In the slice near the diaphragm, the distribution of tidal gas differed between APRV with spontaneous breathing and APRV without spontaneous breathing (*P *< 0.01, Figure 2). Improved ventilation was observed during APRV with spontaneous breathing in both nondependent and dependent ROIs (*P *< 0.05). Differences between dependent and nondependent lung regions in the apical slices were not modified by spontaneous breathing activity (Table 1).

**Figure 3:**
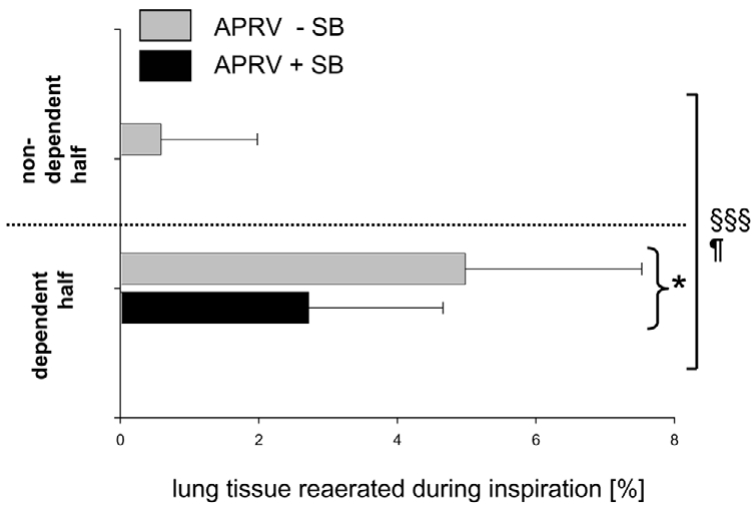
Regional distribution of tidal cyclic collapse. Tidal changes in nonaerated tissue (tidal recruitment) in diaphragmatic slices between a nondependent region of interest (ROI) and a dependent ROI during airway pressure release ventilation (APRV) with or without spontaneous breathing (+SB/-SB). Data presented as the percentage (mean ± standard error of the mean) of the total lung volume (gas and tissue) of voxels for each ROI. ^§§§^*P *< 0.001, dependent ROI versus nondependent ROI. ^¶^*P *< 0.05, APRV +SB versus APRV -SB. *P < 0.05 between ventilatory groups (*post hoc *results given only if significant).

An increase in nonaerated tissue during expiration (tidal collapse) was observed most notably in dependent diaphragmatic lung regions, and was twice as high in the absence of spontaneous breathing as in the presence of spontaneous breathing (*P *< 0.05, Figure 3). No ventilatory-mode-dependent differences for tidal recruitment and collapse were observed in the apical slice (Table 1).

No collapsed lung was observed in nondependent diaphragmatic lung regions at end-inspiration in either ventilatory group. In corresponding dependent diaphragmatic lung regions, end-inspiratory collapse amounted to 14 ± 17% versus 34 ± 18% (*P *< 0.05) during APRV with spontaneous breathing as compared with during APRV without spontaneous breathing.

### Regional attenuation distributions at end-expiration

Analyses of attenuation distributions of apical and diaphragmatic ROIs are presented in Table 2. The nondependent ROI close to the diaphragm included mainly voxels with normal gas content. The overall attenuation distribution was significantly influenced by the ventilatory mode (*P *< 0.01), but *post hoc *testing revealed no difference between single HU ranges. The dependent ROI was dominated by poorly and nonaerated tissue, and the attenuation distribution was also modified by the ventilatory mode (*P *< 0.001). Spontaneous breathing resulted in a larger amount of normally aerated tissue (*P *< 0.01) and in fewer nonaerated voxels (*P *< 0.05).

In the apical slice the interaction of the ventilatory mode and attenuation distribution was significant for nondependent ROIs (*P *< 0.01), but the differences between single HU ranges did not reach statistical significance.

### Regional end-expiratory distribution of gas and nonaerated tissue

**Figure 4:**
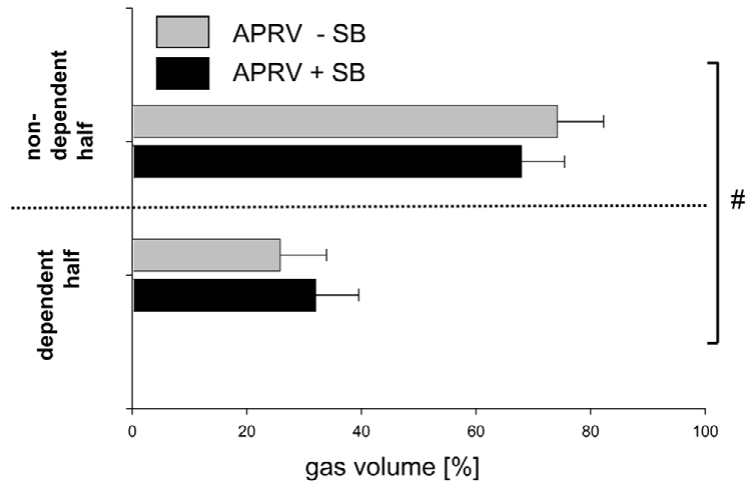
Regional distribution of end-expiratory gas volume. End-expiratory distribution of gas in diaphragmatic slices between a nondependent region of interest (ROI) and a dependent ROI during airway pressure release ventilation (APRV) with or without spontaneous breathing (+SB/-SB). Data presented as the percentage (mean ± standard error of the mean) of the total gas volume of voxels for each ROI. n.s., not significant. ^#^*P *< 0.05, interaction of factor ROI with factor ventilatory mode, suggesting that the ventilatory mode has a significant influence on the regional distribution of end-expiratory gas volume and nonaerated lung tissue.

**Figure 5:**
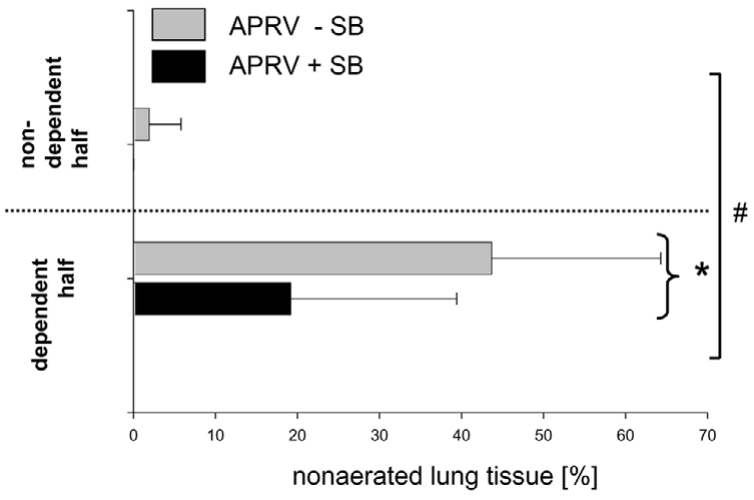
Regional distribution of nonaerated lung tissue. End-expiratory distribution of nonaerated tissue in diaphragmatic slices between a nondependent region of interest (ROI) and a dependent ROI during airway pressure release ventilation (APRV) with or without spontaneous breathing (+SB/-SB). Data presented as the percentage (mean ± standard error of the mean) of the total lung volume (gas and tissue) of voxels for each ROI. n.s., not significant. ^#^*P *< 0.05, interaction of factor ROI with factor ventilatory mode, suggesting that the ventilatory mode has a significant influence on the regional distribution of end-expiratory gas volume and nonaerated lung tissue. *P < 0.05 between ventilatory groups (*post hoc *results given only if significant).

The EELV previously reported from static spiral CT of the whole lung was significantly higher with spontaneous breathing (752 ± 203 ml) as compared with that without spontaneous breathing (353 ± 104 ml; *P *< 0.001) (see also Additional File 1, table III, data kindly reproduced with permission of Lippincott, Williams and Wilkins, Baltimore, MD, USA) [10]. Aeration of the dependent lung region close to the diaphragm was better when spontaneous breathing was allowed during APRV (*P *< 0.05 for the interaction ventilator mode and lung region; Figure 4). Aeration of the nondependent lung region was favored by APRV without spontaneous breathing (*P *< 0.05 for the interaction ventilator mode and lung region; Figure 4). In the dependent lung region, the amount of nonaerated lung tissue was more than twice as large in the animals that did not breathe spontaneously when compared with those that did (*P *< 0.05, Figure 5). No significant differences between ventilator modes were observed in the apical slice (Table 1).

## Discussion

The main findings of this dynamic CT study in oleic-acid-induced lung injury were that spontaneous breathing during APRV is associated with improved tidal ventilation and end-expiratory aeration of dependent juxtadiaphragmatic lung zones and is associated with less tidal alveolar recruitment. The end-inspiratory loss of alveolar aeration, however, was less extensive when spontaneous breathing was allowed during APRV.

### Methodological aspects and limitations of the model

In contrast to endotoxin or lavage models, oleic-acid-induced lung injury is characterized by both inflammation and structural lung damage [20,21] associated with a certain amount of alveolar edema depending on the amount of injury. Whereas lavage models tend to lead to spontaneous recovery of pulmonary function over time, oleic-acid-induced injury produces an injury that is stable in terms of gas exchange impairment for several hours [22].

One could assume that it is necessary to investigate exactly the same lung tissue in both end-inspiratory and end-expiratory images to estimate the regional distribution of tidal ventilation, especially in an inhomogeneous model of lung injury. In a previous investigation comparing different techniques of lung CT in the same experimental model we demonstrated that one single slice is representative for at least three neighbored slices (each 8 mm apart) [23]. The neighboring slices showed no significant differences for any range of attenuation, and this result was not modified by any additional factor such as the ventilatory mode, respiratory phase or scanning technique (spiral or dynamic CT) [23]. In the present analyses ROI sizes appear large enough to minimize effects of transverse movement of the lung tissue. In summary, the technique used in this model of acute lung injury allows one to investigate regional changes of gas content and nonaerated lung tissue during tidal ventilation, knowing that the lung regions investigated during end-inspiration and end-expiration are not necessarily exactly identical.

We used an external PEEP of 5 cmH_2_O in this study, which has been used in previous animal studies [7,8]. This PEEP level in combination with controlled mechanical ventilation was obviously not able to restore the EELV after induction of mild to moderate lung injury in our model [10]. Although no widely accepted strategy to optimize the PEEP in pigs exists, higher PEEP levels might have favored restoration of the EELV [24,25] and might have prevented cyclic alveolar collapse. However, the aim of this study was not to compare different alveolar recruitment strategies but to study the effects of unrestricted spontaneous breathing on lung aeration, which required comparable PEEP levels in both treatment groups. Our study design even resulted in comparable end-inspiratory and mean airway pressures between the groups (see Additional File 1, table III, data kindly reproduced with permission of Lippincott, Williams and Wilkins, Baltimore, MD, USA) [10].

A minority of pigs randomized to receive APRV without spontaneous breathing required neuromuscular blockade to suppress spontaneous breathing. Neuromuscular blockade did not affect gas exchange in patients with severe acute respiratory distress syndrome who had been rendered apneic by lowering the PaCO_2 _[6]. The use of neuromuscular blockade in some animals to guarantee controlled mechanical ventilation therefore cannot explain entirely the changes in cardiopulmonary function and ventilation distribution observed with persisting spontaneous breathing during APRV.

### Tidal ventilation and recruitment

An inhomogeneous distribution of tidal ventilation, as shown earlier in patients with ALI [26-29], may be explained by regional differences in transpulmonary pressures. Transpulmonary pressure is subject to a cephalocaudal gradient, with lower transpulmonary pressures in the caudal parts of the lungs than in the cephalad parts. Mechanisms include the transmission of abdominal pressure to the thoracic cavity [30], an effect that may decrease from the base to the apex, as well as compression of lung tissue by the heart [31]. We observed a redistribution of tidal and end-expiratory volumes to dependent lung regions close to the diaphragm and observed a reduced non-aerated lung volume with spontaneous breathing (Figures 2 and 4). It has been shown that spontaneous diaphragmatic movements are greatest in the posterior parts of the chest [13], which can be partially explained by the location of the muscular parts of the diaphragm preferentially in the lateral and posterior sections and more distended muscle fibers. This should result in locally increased transpulmonary pressures [12], in alveolar recruitment [32], and in improved ventilation of dependent regions close to the diaphragm. In contrast, differences in tidal volume distribution between dependent and nondependent apical lung regions were not significantly influenced by the ventilator mode (Table 1), supporting the idea that diaphragmatic activity is the most important factor influencing ventilation and aeration distribution.

The tidal recruitment was lower with spontaneous breathing. While tidal recruitment was observed during both ventilatory modes, it was more than twice as high without spontaneous breathing in the dependent lung regions near the diaphragm (Figure 3). This cyclic alveolar collapse and reopening causes shear forces with transmural pressures of up to 100 cmH_2_O applied to lung cells [33]. Cyclic opening of lung units during inspiration and closure of lung units during expiration causes mechanical stress, and may contribute to ventilator-associated lung injury including aggravation of pulmonary and systemic inflammation [11,34,35]. We did not measure markers for ventilator-associated lung injury, however, and therefore cannot conclude on the basis of the present data that spontaneous breathing during APRV would prevent or alleviate ventilator-associated lung injury.

Despite the larger amount of tidal recruitment, consolidated lung tissue at end-inspiration was still more pronounced during APRV without spontaneous breathing as compared with during APRV with spontaneous breathing, suggesting that the presence of both distended and collapsed (or consolidated) lung regions was more probable in the absence of spontaneous breathing. An external PEEP higher than 5 cmH_2_O, as used in this study, might have reduced the cyclic alveolar collapse during controlled mechanical ventilation.

### Spontaneous breathing and regional end-expiratory distribution of gas and nonaerated tissue

Previous data from static spiral CT studies of the whole lung have demonstrated that spontaneous breathing results in an increased EELV and in an improvement in arterial blood oxygenation and cardiac output, both contributing to improved oxygen delivery in the same animals [10] (see also Additional File 1, table III, data kindly reproduced with permission of Lippincott, Williams and Wilkins, Baltimore, MD, USA). These quantitative aeration differences associated with spontaneous breathing are extended by the present regional analyses of attenuation distribution (Figure 1 and Table 2), suggesting that an improved EELV is mostly due to alveolar recruitment in dependent juxtadiaphragmatic lung regions with spontaneous breathing and improved regional aeration in dependent lung regions (qualitative aeration differences). These observations may also be explained by higher transpulmonary pressures in dependent juxtadiaphragmatic lung regions, as already discussed. Our data also confirm recent data from single photon emission tomography studies in the same animal model showing not only improvement of ventilation in dependent lung areas close to the diaphragm, but also improvement in regional perfusion of these areas with spontaneous breathing activity [36].

## Conclusion

Results of this study show that spontaneous breathing with APRV promotes alveolar recruitment mainly in dependent, juxtadiaphragmatic lung regions. This results not only in improved end-expiratory aeration, but also in redistribution of the tidal ventilation to dependent lung zones. In addition, spontaneous breathing with APRV countered the undesirable cyclic alveolar collapse in dependent lung regions that can contribute to ventilator-associated lung injury. These data support the hypothesis that active contractions of the diaphragm are a major factor explaining the improved oxygenation by reduction of an intrapulmonary shunt observed in animal models and in patients with ALI. Our data further support the clinical concept to allow, rather than suppress, spontaneous breathing activity in ALI.

## Key messages

• 	In pigs with oleic-acid-induced lung injury, spontaneous breathing with airway pressure release ventilation redistributes tidal ventilation to dependent lung regions close to the diaphragm.

• 	Cyclic alveolar collapse during tidal ventilation, which is considered a risk factor for ventilation-associated lung injury, occurred less in pigs breathing spontaneously.

• 	Previously observed improvement in end-expiratory lung volume in the same animals is explained by alveolar recruitment and redistribution of aeration mainly to dependent juxtadiaphragmatic lung regions.

## Abbreviations

ALI = acute lung injury; APRV = airway pressure release ventilation; CT = computed tomography; EELV = end-expiratory lung volume; FiO_2 _= fraction of inspired oxygen; HU = Hounsfield units; PaCO_2 _= arterial carbon dioxide tension; PEEP = positive end-expiratory pressure; ROI = region of interest.

## Competing interests

The authors declare that they have no competing interests

## Authors' contributions

HW conceived of the study, participated in its design and coordination, performed measurements, and wrote the manuscript. JZ participated in the design of the study, and performed measurements and the statistical analysis. PN conceived of the study, participated in its design and coordination, and performed measurements. TM performed measurements, performed the CT analysis, participated in the study design, and helped draft the manuscript. AM participated in the study design and coordination, and organized the CT measurements. CP conceived of the study, participated in its design and coordination, and helped draft the manuscript. GH conceived of the study, participated in its design and coordination, and revised the manuscript.

## Supplementary Material

Additional File 1 Condensed tables from an online supplement presenting cardiorespiratory effects of the same pigs during APRV with and without spontaneous breathing recorded during a different CT study. Data kindly reproduced with permission of Lippincott, Williams and Wilkins, Baltimore, MD, USA. Originally published as: Wrigge H, Zinserling J, Neumann P, Defosse J, Magnusson A, Putensen C, *et al*.: **Spontaneous breathing improves lung aeration in oleic acid-induced lung injury. ***Anesthesiology *2003, **99:**376-384. Click here for file

## Figures and Tables

**Table 1:** Distribution of tidal ventilation, tidal recruitment gas and nonaerated lung tissue in apical slice

Region	Mode	Tidal ventilation (% of tidal volume)	Tidal recruitment (% of total lung volume)	Gas volume (% of contained gas)	Nonaerated lung tissue (% of total lung volume)
Nondependent	SB-	46 ± 13	0.1 ± 0.2	56 ± 12	0.3 ± 0.6
	SB+	33 ± 31	0.0 ± 0.2	56 ± 6	0.4 ± 0.7
Dependent	SB-	54 ± 13	1.7 ± 1.9	44 ± 12	13.8 ± 14.1
	SB+	67 ± 31	0.5 ± 1.7	44 ± 6	8.6 ± 12.3
Interaction, region × ventilatory mode		ns	ns	ns	ns

Dependent and nondependent region of interest on transverse computed tomography scan (see Figure 1, center for details). SB-, airway pressure release ventilation without spontaneous breathing; SB+, airway pressure release ventilation with spontaneous breathing; ns, not significant.

**Table 2:** Regional densitometries at end-expiration: apical and diaphragmatic slice

Region	Mode	Hyperinflation (%)	Normal aeration (%)	Poor aeration (%)	No aeration (%)	Interaction, HU range × ventilatory mode
Apex						
Nondependent	SB-	0 ± 0	73 ± 17	26 ± 17	1 ± 1	*P *< 0.01
	SB+	0 ± 0	85 ± 11	15 ± 11	0 ± 1	
Dependent	SB-	0 ± 0	33 ± 19	50 ± 14	17 ± 18	ns
	SB+	0 ± 0	48 ± 33	35 ± 21	16 ± 23	
Diaphragm						
Nondependent	SB-	0 ± 0	73 ± 23	25 ± 19	2 ± 5	*P *< 0.01
	SB+	0 ± 0	88 ± 11	12 ± 11	0 ± 0	
Dependent	SB-	0 ± 0	9 ± 9	43 ± 15	47 ± 20	*P *< 0.001
	SB+	0 ± 0	25 ± 15	52 ± 14	23 ± 22	

Data presented as mean ± standard deviation. Amount of hyperinflated, normally aerated, poorly aerated and nonaerated tissue (% of total lung volume) in dependent and nondependent regions of interest on transverse computed tomography scans (see Figure 1 for details). SB-, airway pressure release ventilation without spontaneous breathing; SB+, airway pressure release ventilation with spontaneous breathing; ns, not significant; HU, Hounsfield unit.
