# Supplementary material for: Spontaneous breathing with airway pressure release ventilation favors ventilation in dependent lung regions and counters cyclic alveolar collapse in oleic-acid-induced lung injury: a randomized controlled computed tomography trial
Source: Crit Care. 2005 Nov 16;9(6):R780–9. doi: 10.1186/cc3908 (PMC1414014; doi:10.1186/cc3908)
Supplement: Additional File 1 — Condensed tables from an online supplement presenting cardiorespiratory effects of the same pigs during APRV with and without spontaneous breathing recorded during a different CT study. Data kindly reproduced with permission of Lippincott, Williams and Wilkins, Baltimore, MD, USA. Originally published as: Wrigge H, Zinserling J, Neumann P, Defosse J, Magnusson A, Putensen C, et al.: Spontaneous breathing improves lung aeration in oleic acid-induced lung injury. Anesthesiology 2003, 99:376-384. [file cc3908-S1.doc]

# ONLINE SUPPLEMENT

Data kindly reproduced with permission of Lippincott, Williams and Wilkins, Baltimore, MD, USA.

**Table I Hemodynamics^1)^**

| Parameter | Group |  |  | Lung  Injury |  | 2 h  Treatment |  | 4 h  Treatment |  | Time | Mode | Interaction |
| --- | --- | --- | --- | --- | --- | --- | --- | --- | --- | --- | --- | --- |
|  |  |  |  |  |  |  |  |  |  |  |  |  |
| HR | SB - |  |  | 95 ± 15 |  | 98 ± 15 |  | 98 ± 21 |  |  |  |  |
| [bpm] |  |  |  |  |  |  |  |  |  |  |  |  |
|  | SB + |  |  | 93 ± 20 |  | 102 ± 20 |  | 105 ± 18 |  |  |  |  |
|  |  |  |  |  |  |  |  |  |  |  |  |  |
| MAP | SB - |  |  | 86 ± 10 |  | 81 ± 12 |  | 81 ± 15 |  |  |  |  |
| [mmHg] |  |  |  |  |  |  |  |  |  |  |  |  |
|  | SB + |  |  | 87 ± 12 |  | 90 ± 19 |  | 92 ± 17 |  |  |  |  |
|  |  |  |  |  |  |  |  |  |  |  |  |  |
| CVP | SB - |  |  | 11 ± 2 |  | 11 ± 2 |  | 10 ± 4 |  |  |  |  |
| [mmHg] |  |  |  |  |  |  |  |  |  | * |  |  |
|  | SB + |  |  | 11 ± 2 |  | 10 ± 1 |  | 10 ±2 |  |  |  |  |
|  |  |  |  |  |  |  |  |  |  |  |  |  |
| SVR | SB - |  |  | 1610 ± 353 |  | 1538 ± 377 |  | 1593 ± 383 |  |  |  |  |
| [dyn•s•cm^-5^] |  |  |  |  |  |  |  |  |  |  |  |  |
|  | SB + |  |  | 1553 ± 314 |  | 1493 ± 349 |  | 1488 ± 352 |  |  |  |  |
|  |  |  |  |  |  |  |  |  |  |  |  |  |
| MPAP | SB - |  |  | 34 ± 5 |  | 33 ± 6 |  | 33 ± 6 |  |  |  |  |
| [mmHg] |  |  |  |  |  |  |  |  |  | *** |  | * |
|  | SB + |  |  | 35 ± 6 |  | 31 ± 5 ††† |  | 30 ± 6 ††† |  |  |  |  |
|  |  |  |  |  |  |  |  |  |  |  |  |  |
| PVR | SB - |  |  | 480 ± 136 |  | 467 ± 138 |  | 485 ± 142 |  |  |  |  |
| [dyn•s•cm^-5^] |  |  |  |  |  |  |  |  |  |  |  |  |
|  | SB + |  |  | 464 ± 95 |  | 407 ± 113 |  | 394 ± 135 |  |  |  |  |
|  |  |  |  |  |  |  |  |  |  |  |  |  |
| CO | SB - |  |  | 3.8 ± 0.6 |  | 3.8 ± 0.9 |  | 3.8 ± 1.2 |  |  |  |  |
| [l/min] |  |  |  |  |  |  |  |  |  |  |  | * |
|  | SB + |  |  | 3.9 ± 0.9 |  | 4.4 ± 0.9 |  | 4.5 ± 0.9 † |  |  |  |  |
|  |  |  |  |  |  |  |  |  |  |  |  |  |
| ITBV | SB - |  |  | 615 ± 64 |  | 601 ± 55 |  | 588 ± 81 |  |  |  |  |
| [ml] |  |  |  |  |  |  |  |  |  |  |  |  |
|  | SB + |  |  | 634 ± 45 |  | 637 ± 55 |  | 644 ± 57 |  |  |  |  |

SB-/SB+: APRV *without*/*with* spontaneous breathing, HR: heart rate, MAP: mean arterial blood pressure, CVP: central venous blood pressure, SVR: systemic vascular resistance, MPAP: mean pulmonary artery blood pressure, PVR: pulmonary vascular resistance, CO: cardiac output, ITBV: intrathoracic blood volume. Baseline was only tested against Lung Injury. Post hoc testing was always performed if a significant F ratio for a factor or the interaction of factors was obtained by repeated measures analysis of variance (* p<0.05, ** p<0.01, *** p<0.001), but only significant differences are marked: † p<0.05, †† p<0.01, ††† p<0.001 for within group differences, and ‡ p<0.05 for between group differences (post hoc Tukey´s multiple comparison test).

^1)^ These data have been reported previously (Wrigge H. et al. Anesthesiology 2003; 99:376-84).

**Table II Oxygenation^1)^**

| Parameter | Group |  |  | Lung  Injury |  | 2 h  Treatment |  | 4 h  Treatment |  | Time | Mode | Interaction |
| --- | --- | --- | --- | --- | --- | --- | --- | --- | --- | --- | --- | --- |
|  |  |  |  |  |  |  |  |  |  |  |  |  |
| PaO_2_ | SB - |  |  | 115 ± 32 |  | 90 ± 37 |  | 91 ± 50 |  |  |  |  |
| [mmHg] |  |  |  |  |  |  |  |  |  |  |  | ** |
|  | SB + |  |  | 104 ± 41 |  | 110 ± 47 |  | 144 ± 65 † |  |  |  |  |
|  |  |  |  |  |  |  |  |  |  |  |  |  |
| SaO_2_ | SB - |  |  | 95.9 ± 2.3 |  | 88.8 ± 11.1 |  | 84.0 ± 13.4 †† |  |  |  |  |
| [%] |  |  |  |  |  |  |  |  |  | * |  | * |
|  | SB + |  |  | 91.5 ± 9.9 |  | 90.9 ± 9.6 |  | 91.3 ± 11.3 |  |  |  |  |
|  |  |  |  |  |  |  |  |  |  |  |  |  |
|  | SB - |  |  | 374 ± 64 |  | 345 ± 84 |  | 339 ± 98 |  |  |  |  |
| [ml/min] |  |  |  |  |  |  |  |  |  |  |  | * |
|  | SB + |  |  | 365 ± 93 |  | 409 ± 111 |  | 438 ± 115 |  |  |  |  |
|  |  |  |  |  |  |  |  |  |  |  |  |  |
|  | SB - |  |  | 185 ± 36 |  | 172 ± 42 |  | 160 ± 41 |  |  |  |  |
| [ml/min] |  |  |  |  |  |  |  |  |  |  |  | ** |
|  | SB + |  |  | 172 ±14 |  | 181 ± 29 |  | 186 ± 32 |  |  |  |  |
|  |  |  |  |  |  |  |  |  |  |  |  |  |
| SvO_2_ | SB - |  |  | 48.3 ± 7.8 |  | 44.3 ± 12.6 |  | 43.0 ± 11.8 |  |  |  |  |
| [%] |  |  |  |  |  |  |  |  |  |  |  | * |
|  | SB + |  |  | 46.6 ± 13.7 |  | 49.3 ± 10.6 |  | 55.3 ± 12.1 |  |  |  |  |
|  |  |  |  |  |  |  |  |  |  |  |  |  |
| Qva/Qt | SB - |  |  | 14.4 ± 3.8 |  | 24.2 ± 13.4 |  | 30.8 ± 18.4 †† |  |  |  |  |
| [%] |  |  |  |  |  |  |  |  |  | * |  | * |
|  | SB + |  |  | 21.1 ± 13.5 |  | 22.7 ± 14.6 |  | 21.0 ± 10.9 |  |  |  |  |

SB-/SB+: APRV *without*/*with* spontaneous breathing, PaO_2_: arterial oxygen partial pressure, SaO_2_: arterial oxygen saturation, $\dot{D}O_{2}$: oxygen delivery, $\dot{V}O_{2}$: oxygen consumption, SvO_2_: venous oxygen saturation, : venous admixture. Baseline was only tested against Lung Injury. Post hoc testing was always performed if a significant F ratio for a factor or the interaction of factors was obtained by repeated measures analysis of variance (* p<0.05, ** p<0.01, *** p<0.001), but only significant differences are marked: † p<0.05, †† p<0.01, ††† p<0.001 for within group differences, and ‡ p<0.05 for between group differences (post hoc Tukey´s multiple comparison test).

^1)^ These data have been reported previously (Wrigge H. et al. Anesthesiology 2003; 99:376-84).

**Table III Ventilation^1)^**

| Parameter | Group |  |  | Lung  Injury |  | 2 h  Treatment |  | 4 h  Treatment |  | Time | Mode | Interaction |
| --- | --- | --- | --- | --- | --- | --- | --- | --- | --- | --- | --- | --- |
|  |  |  |  |  |  |  |  |  |  |  |  |  |
| RR | SB - |  |  | 29 ± 3 |  | 32 ± 3 |  | 34 ± 5 |  |  |  |  |
| [1/min] |  |  |  |  |  | ‡ |  |  |  | *** |  | * |
|  | SB + |  |  | 30 ± 0 |  | 41 ±5 |  | 39 ± 9 |  |  |  |  |
|  |  |  |  |  |  |  |  |  |  |  |  |  |
| V_T_ | SB - |  |  | 269 ± 62 |  | 237 ± 43 |  | 231 ± 48 |  |  |  |  |
| [ml] |  |  |  |  |  |  |  |  |  | *** |  |  |
|  | SB + |  |  | 293 ± 64 |  | 208 ± 35 |  | 234 ± 56 |  |  |  |  |
|  |  |  |  |  |  |  |  |  |  |  |  |  |
| V_E_ | SB - |  |  | 8.3 ± 1.9 |  | 8.1 ± 1.6 |  | 8.0 ± 1.6 |  |  |  |  |
| [l] |  |  |  |  |  |  |  |  |  |  |  |  |
|  | SB + |  |  | 9.4 ± 1.1 |  | 8.5 ± 1.2 |  | 8.7 ± 1.6 |  |  |  |  |
|  |  |  |  |  |  |  |  |  |  |  |  |  |
| PaCO_2_ | SB - |  |  | 52 ± 7 |  | 57 ± 7 |  | 56 ±15 |  |  |  |  |
| [mmHg] |  |  |  |  |  |  |  |  |  |  |  |  |
|  | SB + |  |  | 52 ±12 |  | 59 ± 15 |  | 57 ± 16 |  |  |  |  |
|  |  |  |  |  |  |  |  |  |  |  |  |  |
| Ti | SB - |  |  | 1.2 ± 0.2 |  | 1.1 ± 0.3 |  | 1.0 ± 0.1 |  |  |  |  |
| [s] |  |  |  |  |  |  |  |  |  | * |  |  |
|  | SB + |  |  | 1.2 ± 0.2 |  | 1.0 ± 0.4 |  | 0.9 ± 0.3 |  |  |  |  |
|  |  |  |  |  |  |  |  |  |  |  |  |  |
| Te | SB - |  |  | 1.0 ± 0.3 |  | 0.8 ± 0.3 |  | 0.9 ± 0.2 |  |  |  |  |
| [s] |  |  |  |  |  |  |  |  |  | * |  |  |
|  | SB + |  |  | 1.0 ± 0.3 |  | 0.6 ± 0.2 |  | 0.7 ± 0.3 |  |  |  |  |
|  |  |  |  |  |  |  |  |  |  |  |  |  |
| P_aw.ei_ | SB - |  |  | 24.4 ± 3.9 |  | 23.6 ± 3.7 |  | 24.0 ± 3.8 |  |  |  |  |
| [cmH_2_O] |  |  |  |  |  |  |  |  |  |  |  |  |
|  | SB + |  |  | 24.7 ± 4.2 |  | 24.7 ± 4.2 |  | 24.8 ± 4.0 |  |  |  |  |
|  |  |  |  |  |  |  |  |  |  |  |  |  |
| P_aw.mean_ | SB - |  |  | 14.1 ± 3.0 |  | 14.2 ± 2.1 |  | 13.8 ± 2.3 |  |  |  |  |
| [cmH_2_O] |  |  |  |  |  |  |  |  |  |  |  |  |
|  | SB + |  |  | 14.6 ± 2.4 |  | 14.8 ± 3.0 |  | 14.6 ± 2.5 |  |  |  |  |
|  |  |  |  |  |  |  |  |  |  |  |  |  |
| P_trans.mean_ | SB - |  |  | 7.3 ± 3.3 |  | 7.2 ± 2.8 |  | 6.8 ± 2.8 |  |  |  |  |
| [cmH_2_O] |  |  |  |  |  |  |  |  |  |  |  |  |
|  | SB + |  |  | 8.1 ± 2.2 |  | 8.8 ± 3.0 |  | 8.5 ± 3.7 |  |  |  |  |
|  |  |  |  |  |  |  |  |  |  |  |  |  |
| EELV | SB - |  |  | 387 ± 44 |  | 411 ± 165 |  | 384 ± 148 |  |  |  |  |
| [ml] |  |  |  |  |  |  |  | ‡ |  | *** | ** | *** |
|  | SB + |  |  | 416 ± 44 |  | 652 ± 200† |  | 786 ± 320†† |  |  |  |  |

All values are means ± SD, including spontaneous breaths when present. SB-/SB+: APRV *without*/*with* spontaneous breathing, RR: respiratory rate, V_T_: tidal volume, V_E_: minute ventilation, PaCO_2_: arterial carbondioxid partial pressure, Ti: inspiratory time, Te: expiratory time, P_aw.ei_: end-inspiratory airway pressure, P_aw.mean_: mean airway pressure, P_trans.mean_: mean transpulmonary pressure, EELV: end-expiratory lung volume measured by nitrogen washout. Post hoc testing was always performed if a significant F ratio for a factor or the interaction of factors was obtained by repeated measures analysis of variance (* p<0.05, ** p<0.01, *** p<0.001), but only significant differences are marked: † p<0.05, †† p<0.01 for within group differences, and ‡ p<0.05 for between group differences (post hoc Tukey´s multiple comparison test).

^1)^ These data have been reported previously (Wrigge H. et al. Anesthesiology 2003; 99:376-84).
